# The Effect of PPAR Agonists on the Migration of Mature and Immature Eosinophils

**DOI:** 10.1155/2012/235231

**Published:** 2012-06-28

**Authors:** Steven G. Smith, Haruki Imaoka, Neha Punia, Anam Irshad, Luke L. Janssen, Roma Sehmi, Gail M. Gauvreau

**Affiliations:** ^1^Department of Medicine, McMaster University, Hamilton, ON, Canada L8S 4K1; ^2^Department of Medicine, Kurume University School of Medicine, Kurume, Fukuoka 830-0011, Japan

## Abstract

PPAR**γ** agonists can either enhance or inhibit eosinophil migration, which is a sum of directional migration (chemotaxis) and random cell movement (chemokinesis). To date, the effects of PPAR agonists on chemokinesis have not been examined. This study investigates the effects of PPAR**α**, **δ**, and **γ** agonists on eosinophil migration and chemokinesis. Eosinophils purified from blood of atopic donors were preincubated with rosiglitazone (PPAR**γ** agonist), GW9578 (PPAR**α** agonist), GW501516 (PPAR**δ** agonist), or diluent. The effects of PPAR agonists were examined on eosinophil chemokinesis, eotaxin-induced migration of eosinophils, and migration of IL-5R**α**+ CD34+ cells. Expressions of CCR3, phospho-p38, phospho-ERK, and calcium release were also measured in eosinophils after rosiglitazone treatment. Low concentrations of rosiglitazone, but not GW9578 or GW501516, increased chemokinesis of eosinophils (*P* = 0.0038), and SDF-1**α**-induced migration of immature eosinophils (*P* = 0.0538). Rosiglitazone had an effect on eosinophil calcium flux but had no effect on expression of CCR3 or phosphorylation of p38 or ERK. In contrast, high concentrations of rosiglitazone inhibited eosinophil migration (*P* = 0.0042). The effect of rosiglitazone on eosinophil migration and chemokinesis appears to be through modification of calcium signaling, which alludes to a novel PPAR-mediated mechanism to modulate eosinophil function.

## 1. Introduction

Eosinophils are effector cells which contribute to the pathology of allergic diseases [1]. They are recruited from the blood into inflamed tissue by local release of chemokines [2, 3]. Eotaxin-1, which is one of the most potent eosinophil chemokines, signals through chemokine receptor 3 (CCR3). Novel approaches toward inhibiting migration of effector cells such as eosinophils are being investigated for treatment of allergic asthma.

The peroxisome proliferator-activated receptors (PPARs) are metabolite-activated transcription factors that have been shown to regulate metabolic and inflammatory responses [4]. There are three identified subtypes of PPARs: PPAR*α* [5], PPAR*γ* [6], and PPAR*δ* [7], which have attracted interest as therapeutic targets in lung disease due to their preferential expression on human airway smooth muscle [8] and inflammatory cells [9] and increased expression during inflammatory events [10, 11]. In murine models of allergic asthma, PPAR*α* and PPAR*γ* agonists (rosiglitazone and GW9578, resp.) inhibit eosinophil influx to the lung following airway antigen challenge [12, 13]. The PPAR*γ* agonist was more effective than the PPAR*α* agonist, while the PPAR*δ* agonist had no effect [13].

At low concentrations, however, PPAR agonists have been shown to enhance eosinophil migration *in vitro* [14]. The observed enhancement in eosinophil migration could be due to an increase in chemotaxis or chemokinesis [15], and the current study will compare the effects of low concentrations of the PPAR agonists GW9578, GW501516, and rosiglitazone on eosinophil migration and chemokinesis.

## 2. Materials and Methods

### 2.1. Subjects

Blood was obtained from 10 male and 13 female, nonsmoking, atopic donors aged 19–60 years old. Atopy was confirmed by skin prick testing. Subjects were not currently using steroidal or nonsteroidal anti-inflammatory medications or antihistamines. Samples of cord blood were obtained from the hospital delivery room from 6 individuals whose atopic status and medication use were not determined. The study was approved by the FHS/HHS Research Ethics Board, and subjects gave informed consent to participate.

### 2.2. Eosinophil Purification

One hundred mL of peripheral blood was collected from each subject into sodium heparin vacutainers for *in vitro* experiments. Peripheral blood was diluted with an equal volume of McCoy's 5A (Invitrogen Canada Inc., Burlington, ON, Canada), and eosinophils were purified using an AccuPrep density gradient (Accurate Chemical & Scientific Corporation, Westbury, NY, USA) followed by a MACS column CD16+ neutrophil depletion (Miltenyi Biotec, Auburn, CA, USA). The purity of each eosinophil sample was determined with a CytoPrep stained for a cell differential count (Diff Quik; *Siemens* Healthcare Diagnostics, Deerfield, IL, USA). Light microscopy demonstrated that the eosinophil preparations were >90% pure and the majority of contaminating cells were neutrophils.

Purified eosinophils were resuspended in RPMI complete (10% Fetal Bovine Serum, 1 M HEPES in RPMI 1640) and incubated for 20 minutes with 0.1 nM–100 *μ*M of either a PPAR*α* agonist (GW9578; Cayman Chemical, Ann Arbor, MI, USA), a PPAR*β*/*δ* agonist (GW501516; Axxora LLC, San Diego, CA, USA), or a PPAR*γ* agonist (rosiglitazone; Cayman Chemical, Ann Arbor, MI, USA), all in a final concentration of 0.1% DMSO, or with diluent (RPMI complete in 0.1% DMSO). Treatment with PPAR agonists had no effect on eosinophil viability as determined by trypan blue exclusion, being >98% viable before and after incubation.

### 2.3. Eotaxin-Induced Eosinophil Migration

The migration assay used a 48-well Boyden chamber, following protocols from previous studies on eosinophil migration [16]. The PPAR agonists were left in the cell suspension, and the upper wells of the Boyden chamber were filled with 50 *μ*L of eosinophils at a concentration of 3 × 10^6^ cells/mL. Eotaxin at a concentration of 10 nM (R&D Systems, Minneapolis, MN, USA) in the presence of equivalent concentrations of PPAR agonist (0.1 nM–100 *μ*M) was placed in the lower wells of the microchemotaxis assembly (Neuro Probe Inc., Gaithersburg, MD, USA). The upper and lower wells were separated by a nitrocellulose filter with an 8 *μ*m pore, and the chamber was incubated in high humidity for 90 minutes at 37°C. The chamber was then disassembled, and the nitrocellulose filter was fixed with mercuric chloride overnight and stained with hematoxylin and chromotrope 2R. The filter were dehydrated through graded alcohols followed by a 30-minute xylene treatment, then mounted on a glass slide and coverslipped with Permount (*Fisher Scientific*, Pittsburgh, PA, USA). The number of eosinophils was quantified from 10 random fields at the leading edge, counted from the underside of the filter using a light microscope at 400x magnification.

### 2.4. PPAR Agonist-Induced Eosinophil Chemokinesis

To determine the effects of PPARs on eosinophil chemokinetic responses, eosinophils were incubated with PPAR agonists (0.1 nM–100 *μ*M) for 20 minutes then loaded into the upper chamber with similar concentrations of the PPAR agonist in the lower chambers.

### 2.5. Eosinophil CCR3 Expression

Purified eosinophils were incubated with rosiglitazone at 0.1 nM or 10 nM or diluent for 110 minutes. The cells were then stained for CCR3 surface expression using mouse anti-human Pacific Blue-CD45 (eBioscience, San Diego, CA, USA), FITC-CD16 (Becton-Dickinson Biosciences, Mississauga, ON, Canada), and PE-CCR3 (Medical & Biological Laboratories, Naka-ku, Nagoya, Japan), as well as the isotype control antibodies for CCR3. Cells were acquired with an LSR II flow cytometer (Becton Dickinson Instrument Systems; Becton-Dickinson, Mississauga, ON, Canada) using the FACSDiva software program (Becton-Dickinson Biosciences). Fluorometric compensation was set to minimize autofluorescence, a known issue surrounding eosinophil and flow cytometry [17].

### 2.6. Eosinophil Migration—Signal Transduction Pathways

Purified eosinophils were treated with rosiglitazone at concentrations which was previously reported to induce cell migration (0.1 nM, 10 nM) [14] or with diluent for 110 minutes in the absence or presence of 10 nM eotaxin. After rosiglitazone treatment, eosinophils were lysed and the protein concentration was standardized using a Bradford assay. Phosphorylation of ERK1/2 and p38 was analyzed using signal transduction assay reaction (STAR) ELISA kits (Millipore, Temecula, CA, USA) and quantified by measuring the absorbance at 450 nm using an EL800 plate reader (BioTek Instruments, Winooski, VT, USA).

### 2.7. Eosinophil Progenitor Cell Transwell Migration

Cord blood was diluted with an equal volume of McCoy's 5A (Invitrogen Canada Inc.), and mononuclear cells were purified using an AccuPrep density gradient (Accurate Chemical & Scientific Corporation, Westbury, NY, USA). Non-adherent mononuclear cells (NAMCs) were then resuspended in McCoy's 3+ (McCoy's 5A with 10% fetal bovine serum, 1% penicillin/streptomycin, and 1% 2-mercaptoethanol) and incubated for 2 hours in 5% CO_2_ at 37°C and high humidity to remove monocytes. CD34+ progenitor cells were isolated using a MACS column CD34+ positive selection (Miltenyi Biotec, Auburn, CA, USA). The CD34+ progenitor cell preparations were >90% viable after isolation.

CD34+ progenitor cells were resuspend at 1 × 10^6^ cells/mL in RPMI complete (10% fetal bovine serum, 1 M HEPES in RPMI 1640) with 100–1000 nM of PPAR*α* agonist (GW9578), PPAR*β*/*δ* agonist (GW501516), or PPAR*γ* agonist (rosiglitazone) agonist, all in a final concentration of 0.1% DMSO or diluent (RPMI complete in 0.1% DMSO). SDF-1*α* (R&D Systems) at a concentration of 100 ng/mL in the presence of equivalent concentrations of PPAR agonists (100–1000 nM) was placed in the lower wells of the transwell assembly. The chamber was incubated in high humidity for 18 hours at 37°C. The chamber was then disassembled, and the cells from the lower well were stained for CD34 and IL-5R*α* surface expression using mouse anti-human CD34-APC, CD45-FITC, and PE-IL-5R*α* (Becton-Dickinson Biosciences), as well as the isotype control antibodies for CCR3. Cells were acquired with an LSR II flow cytometer (Becton Dickinson Instrument Systems; Becton-Dickinson) using the FACSDiva software program (Becton-Dickinson Biosciences). Migration was expressed as % of total CD34+ cells plated.

### 2.8. Image Acquisition and Measurement of [Ca^2+^]_*i*_

Intracellular changes in calcium were measured using confocal microscopy, as previously described [18]. Briefly, isolated eosinophils (1 × 10^6^ cells/mL) were loaded for 1 hr on ice with 3.5 *μ*M of a Ca^2+^-sensitive fluorescent probe, fluo-3 AM (Invitrogen Canada) dissolved in dimethyl sulfoxide with 0.01% Pluronic F-127. The eosinophils were then loaded onto a culture dish and placed on the stage of a custom-built confocal microscope equipped with a 20x objective. The bathing solution for all experiments was RPMI, maintained at 37°C, which was exchanged constantly via superfusion throughout the experiment. Eosinophils were then illuminated using 488 nm light from a 20 mW photodiode laser (Coherent Technologies; CA, USA). During the recordings eosinophils were exposed to control (diluent or eotaxin) or treatment (diluent with 100 nM rosiglitazone or eotaxin with 100 nM rosiglitazone). Images (480 × 640 pixels) were collected at 30 Hz with the imaging software “Video Savant” (IO Industries; London, ON, Canada); 9 consecutive frames were then averaged at 1.5 second intervals, giving a final image rate of 0.67 Hz. The image analysis software, “Scion” (Scion Corporation, Frederick, MD, USA), was used to determine the pixel intensity of 10 individual cells for measurement of [Ca^2+^]_*i*_. Fluorescence intensities of the regions of interest were saved and plotted against time. An increase in average fluorescence intensity was interpreted as an increase in [Ca^2+^]_*i*_, and an increase in the average frequency of [Ca^2+^]_*i*_ spikes was interpreted as an increase in [Ca^2+^]_*i*_ oscillations.

### 2.9. Statistical Analysis

All data are expressed as the mean ± standard error unless otherwise stated. Statistical analyses were performed using Prism version 5 (GraphPad Software, La Jolla, CA, USA). Analysis of variance (ANOVA) was used to compare PPAR agonist treatments versus diluent at the various doses, with post hoc Tukey tests for prespecified comparisons. For data not normally distributed, the statistics were performed on the log-transformed data. Statistically significant differences were accepted at *P* < 0.05.

## 3. Results and Discussion

### 3.1. Subjects

Blood donors that were recruited for the study were 31 ± 12 years old with mild blood eosinophilia (3.9 ± 2% eosinophils).

### 3.2. Effects of PPAR Agonists on Eotaxin-Induced Eosinophil Migration

**Figure 1:**
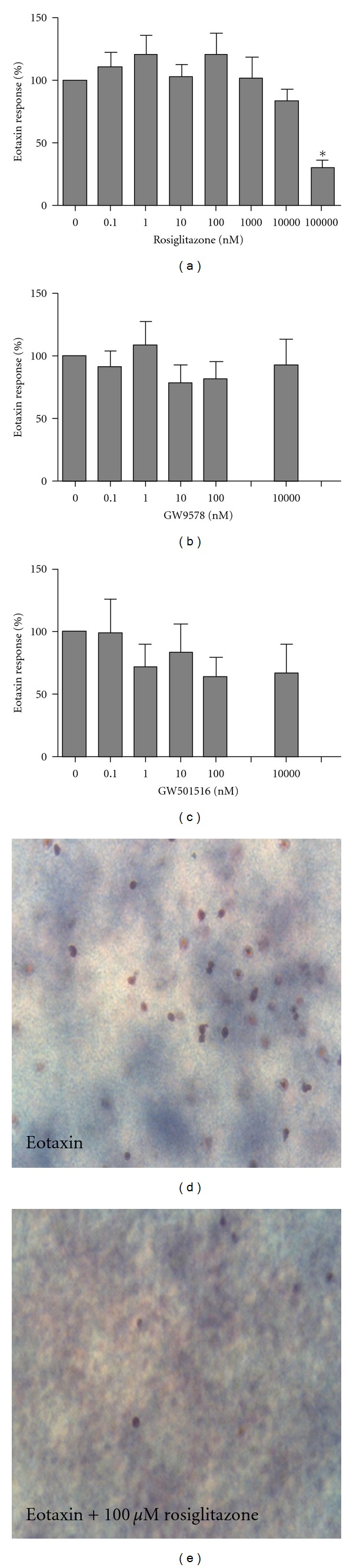
The effect on eotaxin-induced eosinophil migration of agonists to PPAR*γ* ((a) rosiglitazone), PPAR*α* ((b) GW9578), and PPAR*δ* ((c) GW50516) at low (0.1–100 nM; 9 subjects) and high concentrations (1000–100,000 nM; 6 subjects). Representative pictures at 200x magnification of the leading edge of the nitrocellulose filter after incubation with eotaxin (d) and eotaxin with 100 *μ*M rosiglitazone (e). Data are shown as mean ± SEM and expressed as % of the response to eotaxin.

The chemokine eotaxin (10 nM) induced significant eosinophil migration compared to diluent control (242.9 ± 148.6 versus 46.1 ± 68.1 cells/10 HPF, *P* < 0.0001). PPAR agonists were tested at low concentrations (*n* = 9, Figures 1(a), 1(b), and 1(c)) and high concentrations (*n* = 6, Figures 1(a), 1(b), and 1(c)). Preincubation with 100 *μ*M rosiglitazone significantly inhibited eosinophil migration (Figures 1(a) and 1(e); *P* = 0.0042). By contrast, there was no effect of GW9578 (*P* = 0.9) or GW501516 (*P* = 0.3) on eotaxin-induced migration (Figures 1(b) and 1(c)). 

**Figure 2:**
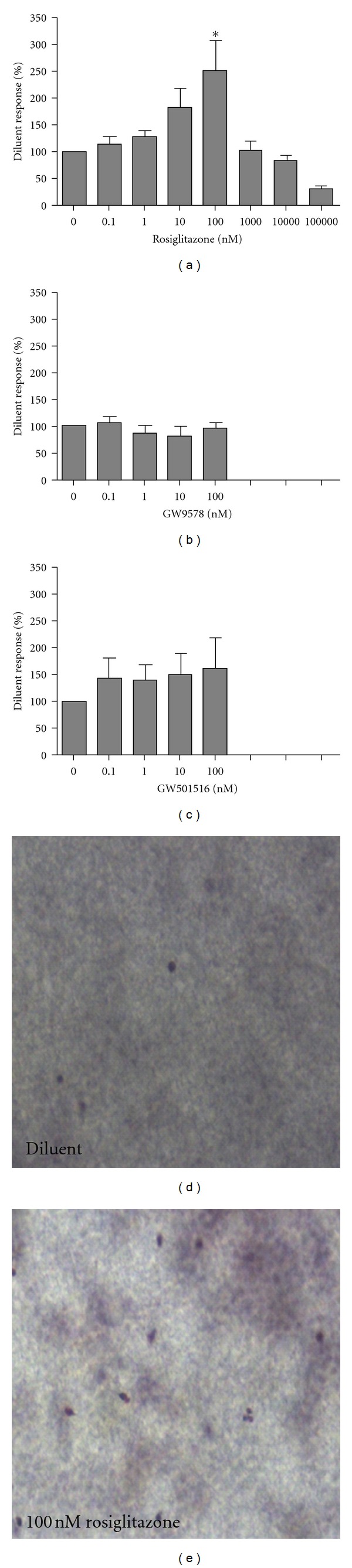
The effect on eosinophil chemokinesis of agonists to PPAR*γ* ((a) rosiglitazone), PPAR*α* ((b) GW9578), and PPAR*δ* ((c) GW50516) at low (0.1–100 nM; 9 subjects) and for PPAR*γ* (a; rosiglitazone) at high concentrations (1000–100,000 nM; 6 subjects). Representative pictures at 200x magnification of the leading edge of the nitrocellulose filter after incubation with diluent (d) and 100 nM rosiglitazone (e). Data are shown as mean ± SEM and expressed as % of the response to diluent.

Rosiglitazone treatment alone at a concentration of 100 nM both above and below the nitrocellulose filter in the micro-Boyden chamber assay significantly increased the eosinophil chemokinesis compared to diluent control (*P* = 0.0038; Figures 2(a) and 2(e)). In contrast, no chemokinetic responses were observed when eosinophils were incubated with equivalent concentrations of GW9578 (PPAR*α* agonist, *P* = 0.9) or GW501516 (PPAR*δ* agonist, *P* = 0.9) above and below the filter (Figures 2(b) and 2(c)), or with a high concentration of rosiglitazone (Figure 2(a), *P* = 0.05).

Cell migration is a multistep process, which involves both chemotaxis and chemokinesis in response to a chemokine [19]. Chemotaxis is defined as directed migration towards a chemokine, whereas chemokinesis is defined as non-directional migration. Chemokinesis, alone, is not sufficient for cell accumulation but may contribute considerably by priming a cell to respond more vigorously to a chemotactic stimuli [20]. Previously it was unknown whether the increase in eotaxin-induced migration by PPAR*γ* agonists was due to chemotaxis, chemokinesis, or both. Although PPAR agonists in this *in vitro* study are used at concentrations similar to the reported EC_50_s those for rosiglitazone, GW9578, and GW501516 are 43 nM, 50 nM, and 1.1 nM, respectively [21–23], this study has determined that the enhanced migration by rosiglitazone is likely due to a chemokinetic effect. Such “priming” of cells may enhance their response to other stimuli, including chemokines such as eotaxin that are found in the microenvironment of allergic tissue, and thus lead to eosinophilia.

### 3.3. Effect of Rosiglitazone on Eosinophil CCR3 Surface Expression and Signaling

Incubation with rosiglitazone at 0.1 and 10 nM had no effect on eosinophil CCR3 surface expression (Table 1). Furthermore, eosinophil incubation with rosiglitazone in the presence or absence of eotaxin had no effect on the level of phosphorylation of ERK1/2 or p38 MAPK (Table 1).

To improve our understanding of how PPAR agonists regulate eotaxin-induced eosinophil migration, we investigated the effects of rosiglitazone on the surface expression and downstream signalling of the eotaxin receptor, CCR3. Consistent with studies of other PPAR*γ* agonists, 15d-PGJ_2_ and troglitazone [14], which were studied at similar concentrations, we observed no effect of rosiglitazone on the level of cell surface expression of CCR3 on eosinophils or on phosphorylation of the downstream signaling molecules ERK1/2 and p38.

### 3.4. Effects of PPAR Agonist on SDF-1*α*-Induced Eosinophil Progenitor Cell Migration

**Figure 3:**
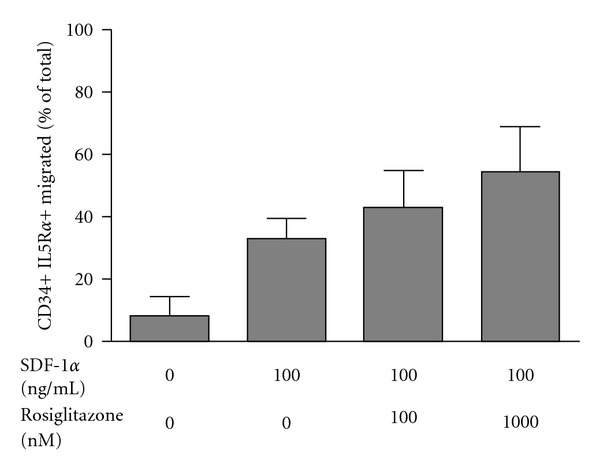
The effect of the PPAR*γ* agonist rosiglitazone on SDF-1*α*-induced migration of IL-5R*α*+ CD34+ cells isolated from cord blood (6 subjects). Data are shown as mean ± SEM and expressed as a % of the total IL-5R*α*+ CD34+ cells.

Compared to diluent control, stromal cell-derived factor-1*α* (100 ng/mL) induced migration of IL-5R*α*+ CD34+ cells isolated from cord blood (8.2 ± 6.2 versus 33.2 ± 6.1% of total cells migrated, *P* = 0.0479). Low concentrations of rosiglitazone increased the migrational response of cord blood-derived IL-5R*α*+ CD34 cells to SDF-1*α* compared to diluent control; however, this change did not reach the level of statistical significance (Figure 3; *P* = 0.054). No changes in migration of IL-5R*α*+ CD34+ cells were observed with equivalent concentrations of GW9578 (PPAR*α* agonist, *P* = 0.8) or GW501516 (PPAR*δ* agonist, *P* = 0.6) (data not shown).

We also examined the effect of PPAR agonists on the immature eosinophil population of IL-5R*α*+ CD34 cells purified from cord blood samples. We demonstrated that migration of these immature eosinophils in response to the potent chemoattractant SDF-1*α* is likewise enhanced by pretreatment of low concentrations of rosiglitazone. This finding is novel and mirrors our and Koybayashi's [14] observations in mature eosinophils, of enhanced migration by low concentrations of PPAR*γ* agonist, with no effect of PPAR*α* and *δ* agonists.

### 3.5. Image Acquisition and Measurement of [Ca^2+^]_*i*_

**Figure 4:**
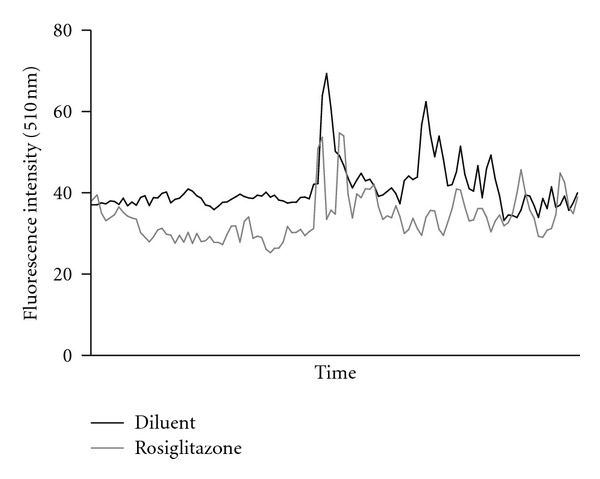
The effect of 100 nM rosiglitazone treatment on the eotaxin-induced increase in  [Ca^2+^]_*i*_, measured by the frequency of calcium oscillations and maximum fluorescence intensity (representative tracing from 3 experiments).

Calcium flux was examined in 3 separate eosinophil preparations. Addition of eotaxin to eosinophil preparations caused an increase in maximum fluorescence intensity (Figure 4; diluent 16.6 ± 6.2 versus eotaxin 66.7 ± 27.4) and the number of calcium oscillations (diluent 0.007 ± 0.004 Hz versus eotaxin 0.038 ± 0.016 Hz). Treatment with 100 nM rosiglitazone consistently reduced the frequency of calcium oscillations observed after the addition of 10 nM eotaxin (control 0.038 ± 0.016 Hz versus treatment 0.016 ± 0.007 Hz). However, no consistent effect of rosiglitazone was observed on the maximum fluorescence intensity in the 3 preparations studied (control 66.7 ± 27.4 versus treatment 58.1 ± 25.2).

Previous studies have shown that pretreatment with a MEK inhibitor or a p38 MAPK inhibitor had no effect on enhanced migration nor did inhibition of the NF-*κ*B pathway or inhibition of genomic transcription with actinomycin D [14]. We and others have shown that PPAR agonists have a modulatory effect on eotaxin-induced calcium mobilization [14], which would suggest that PPAR agonists have downstream targets which have not been identified yet.

## 4. Conclusion

This is the first study to show increased chemokinesis of eosinophils *in vitro* at low concentrations of rosiglitazone, which has a 100-fold higher binding affinity for PPAR*γ* than troglitazone [24]. The results of this study also confirm previous observations showing that high concentrations of a PPAR*γ* agonist inhibit eosinophil migration [24].

We demonstrated that the PPAR*γ* agonist rosiglitazone, at a concentration of 100 nM significantly increased eotaxin-induced eosinophil chemokinesis *in vitro*. This finding supports the data demonstrating increased eosinophil migration reported by Kobayashi et al. [14]. We also demonstrated that rosiglitazone treatment had no effect on the level of cell surface expression of the eotaxin receptor, CCR3, on eosinophils, or on the downstream signalling events following eotaxin/CCR3 binding, such as phosphorylation of ERK1/2 and p38 MAPK, suggesting that the observed effects of rosiglitazone are not related to alterations in signalling through the eotaxin receptor. At these same concentrations there was no effect of the PPAR*α* agonist, GW9578, or the PPAR*δ* agonist, GW501516.

In summary, at low concentrations the PPAR*γ* agonist rosiglitazone enhanced chemokinesis of eosinophils isolated from the peripheral blood of atopic subjects and also enhanced the migration of eosinophil progenitors. This chemokinetic effect may at least partially explain the enhancement of eosinophil migration seen at these concentrations. The enhanced chemokinesis was specific for the PPAR*γ* agonist, as there was no effect of PPAR*α* or PPAR*δ* agonists. In light of our findings that a selective PPAR*γ* agonist can enhance eosinophil chemokinesis at a concentration of 100 nM and decrease eosinophil migration at a concentration of 100 *μ*M, the therapeutic window for PPAR*γ* agonists as an anti-inflammatory therapy is narrow and dosage must be titrated carefully.

## Figures and Tables

**Table 1:** Eosinophil expression of CCR3 and phosphorylation of ERK1/2 and p38 after treatment with the PPAR*γ* agonist rosiglitazone.

		Diluent	0.1 nM	10 nM	*P* value
CCR3 (%)		29.3 ± 7.5	23.5 ± 5.7	19.8 ± 4.7	0.1
CCR3 (MFI)		8.3 ± 4.4	6.8 ± 3.3	5.4 ± 2.8	0.2

Phospho-ERK1/2 (units/mL)	Diluent	3.5 ± 0.7	3.3 ± 0.6	3.3 ± 0.5	0.6
	Eotaxin	3.75 ± 0.2	3.93 ± 0.4	3.95 ± 0.4	0.8

Phospho-p38 (units/mL)	Diluent	3.9 ± 1	4.8 ± 1.3	4.5 ± 1.8	0.9
	Eotaxin	6.7 ± 1.9	6.2 ± 1.5	7.0 ± 1.4	0.8

Data represent mean + SEM.
